# Acute pancreatitis as a complication of epstein-barr virus infection: A case report and narrative review

**DOI:** 10.1016/j.idcr.2025.e02469

**Published:** 2025-12-22

**Authors:** Joe Zako, Gilbert Cornut, Alexandra Monière-Wollank

**Affiliations:** a Faculty of Medicine, Université de Montréal, Montreal, QC, Canada; b Department of Family Medicine and Emergency Medicine, Centre intégré universitaire de santé et services sociaux du Centre-Sud-de-l’Île-de-Montréal, Montréal, QC, Canada; c Department of Microbiology, Infectiology and Immunology, Centre intégré universitaire de santé et services sociaux du Centre-Sud-de-l’Île-de-Montréal, Montréal, QC, Canada

**Keywords:** Epstein–Barr virus, Acute pancreatitis, Case report, Peritonsillar abscess, Narrative review, Infectious mononucleosis

## Abstract

**Background:**

Epstein–Barr virus (EBV) infection is common, but EBV-associated acute pancreatitis is rare and heterogeneous. We describe an adult case and synthesize published cases to inform diagnosis and management.

**Case presentation:**

A 40-year-old man presented with sore throat and mild abdominal pain; examination showed a right peritonsillar abscess with uvular deviation. Laboratory testing revealed lipase 3704 U/L and elevated inflammatory markers; abdominal CT confirmed non-complicated acute pancreatitis. He received incision and drainage of the abscess plus conservative pancreatitis care. Symptoms resolved rapidly, lipase decreased to 352 U/L by day 2, and he was subsequently discharged. EBV serology later confirmed acute infection, and patient was asymptomatic at 2-month follow-up.

**Methods:**

We searched PubMed to August 24, 2025, using (“Epstein-Barr virus” OR “EBV”) AND “pancreatitis,” including human case reports or series of EBV-associated acute pancreatitis. There were no language restrictions.

**Results:**

Of 60 records, 13 cases met criteria. Eight involved adults and most patients were female. Abdominal pain was common, but classic mononucleosis symptoms were infrequently reported. CT was the predominant diagnostic modality. Management was conservative in nearly all reports and antivirals were rarely used. Outcomes were generally favorable, with one fatal case.

**Conclusions:**

EBV should be considered in unexplained acute pancreatitis, particularly when common etiologies are excluded or concurrent EBV features are present. Prognosis is typically good with supportive care, but coexisting risk factors may predispose patients to a more severe or complicated course. Transparent reporting of cofactors will clarify whether EBV acts as an opportunistic trigger or independent cause.

## Highlights

• EBV-associated acute pancreatitis is a rare but recognized complication in both pediatric and adult patients.
• The clinical presentation is heterogeneous and may often lack typical features of infectious mononucleosis.
• Conservative management is usually sufficient, and prognosis is favorable in most cases.
• Coexisting risk factors may lower the threshold for EBV-related pancreatic inflammation.
• Reporting of risk factors could clarify whether EBV acts as an opportunistic trigger or an independent cause of pancreatitis.

## 1 Background

Epstein-Barr Virus (EBV) is a ubiquitous herpesvirus, carried by over 95 % of adults worldwide, with primary infection usually occurring in childhood or adolescence. By infecting host B-lymphocytes and establishing latency in memory B-cells, it persists lifelong and may reactivate in the setting of immunosuppression [1]. Most EBV infections are self-limited and resolve without intervention within a few weeks, with fatigue being the most persistent symptom, but a minority of cases can lead to serious and sometimes life-threatening complications, the most recognized being spontaneous splenic rupture with hemorrhage [1], [2]. Reported complications also include tonsillar edema with airway compromise or bacterial superinfection, acute acalculous cholecystitis, myocarditis, encephalitis and hemophagocytic lymphohistiocytosis [1], [2].

Gastrointestinal involvement is also relatively common, usually in mild form (e.g. transient elevations in liver enzymes), but acute pancreatitis is an exceedingly rare complication of EBV infection. It has been previously documented in the literature, with most cases involving children or young adults following primary infection, but it has also been reported a number of times in an older population [3]. While most cases of acute pancreatitis are gallstone-related or alcohol-induced, infectious causes still account for ∼10 % of cases [4], [5]. The exact pathogenesis of EBV-related pancreatitis is not fully understood; both direct viral injury of pancreatic cells and an indirect, immune-mediated inflammatory mechanism have been proposed [6]. In the following case report and review of the literature, we aim to shed light on this rare complication and highlight the importance of considering EBV in the differential diagnosis of acute pancreatitis, particularly when common etiologies are unlikely or excluded.

## 2 Methods

### 2.1 Case report

Clinical data for this case were obtained from the patient’s medical record, including history, physical examination, laboratory findings, imaging studies, and hospital course. Written informed consent was obtained from the patient for publication of this case report; ethical approval was not required for single-patient case reports at our institution. The report was prepared in accordance with the CARE guidelines [7].

### 2.2 Narrative review

We searched PubMed for articles published up to August 24th, 2025, using the terms (“Epstein-Barr virus” OR “EBV”) AND “pancreatitis”. Only case reports and case series describing human patients with EBV-associated acute pancreatitis were included. Reports of pancreatitis secondary to intra-abdominal EBV-associated tumors (e.g., B-cell lymphoma) were excluded. No restrictions were placed on language of publication.

### 2.3 Case presentation

A 40-year-old obese male presented to the Notre-Dame Hospital emergency department with a sore throat and mild abdominal pain. Past medical history included obstructive sleep apnea, moderately elevated triglycerides (4.13 mmol/L), untreated cutaneous psoriasis and gastro-esophageal reflux disease. At-home medications were pantoprazole as-needed and an 8-month course of weekly semaglutide injections for weight loss. Alcohol consumption averaged 7 drinks per week. On examination, the patient was afebrile but appeared fatigued and tachypneic with a respiratory rate of 23 breaths per minute, oxygen saturation of 93 %, a right tonsillar mass with deviation of the uvula to the left, and mild epigastric tenderness.

**Fig. 1:**
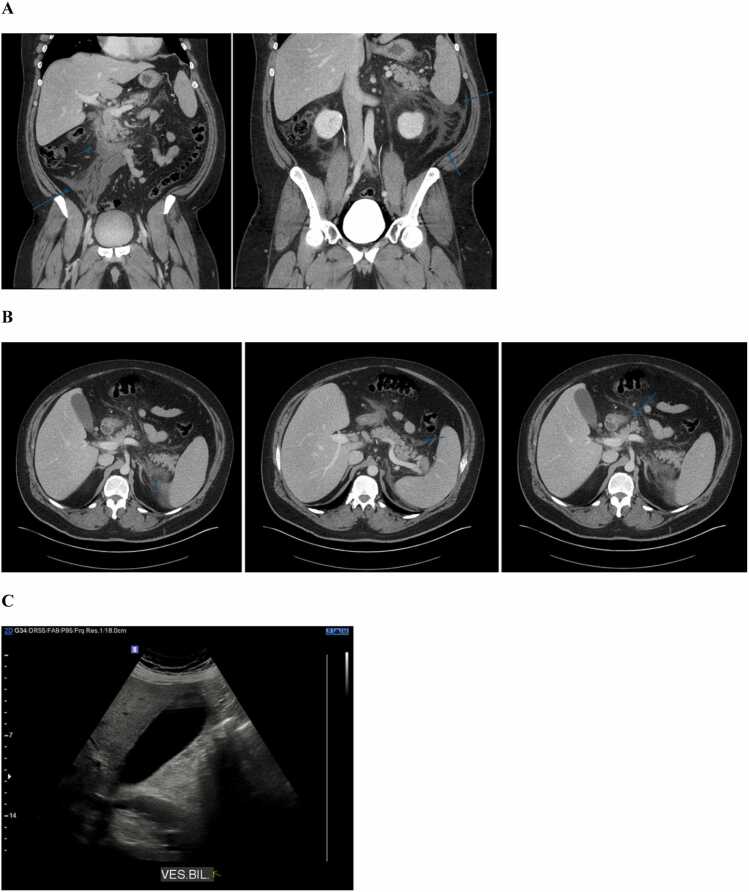
Diagnostic pancreatitis imaging. A Coronal CT view showing fat infiltration in the anterior pararenal space extending into the right iliac fossa and the left flank; B Axial CT view showing peripancreatic fat infiltration, with no venous or arterial thrombosis and no signs of pancreatic necrosis; C Gallbladder ultrasound showing absence of gallstones.

Initial laboratory workup showed a positive mononucleosis spot test and negative rapid streptococcal antigen test. Bloodwork results showed the following: hemoglobin 138 g/L; platelets 215 × 10⁹/L; white blood cells 10.6 × 10⁹/L; C-reactive protein 190 mg/L; aspartate aminotransferase 49 U/L; alanine aminotransferase 120 U/L; total bilirubin 16 µmol/L; and lipase 3704 U/L. Computed tomography (CT) scanning of the neck with contrast demonstrated bilateral tonsillitis with a right peritonsillar abscess (25 × 21 × 32 mm). Abdominal CT with contrast showed non-complicated acute pancreatitis, and ultrasound revealed moderate diffuse hepatic steatosis without gallstones or bile duct dilation (Fig. 1).

The patient was started on intravenous (IV) ceftriaxone, metronidazole, and dexamethasone. An ear, nose, and throat specialist performed incision and drainage of the abscess, after which respiratory symptoms resolved. Antibiotics were later switched to oral clindamycin. The pancreatitis was managed with conservative measures including bed rest, fasting, IV nutritional support, and analgesics. By hospital day 2, abdominal pain had resolved, and lipase decreased to 352 U/L, permitting discharge. Due to laboratory processing times, serology results only became available several days after discharge, confirming acute EBV infection (positive EBV-VCA IgM, negative EBV-EBNA IgG, negative CMV IgG and negative hepatitis A, B, C serologies). The patient was called two months later for follow-up and reported absence of relapse and no residual symptoms of pancreatitis or mononucleosis.

### 2.4 Narrative review

The search terms utilized yielded 60 entries in the PubMed database. Of these entries, 13 publications totalling 13 individual eligible patient cases fit our inclusion criteria [3], [8], [9], [10], [11], [12], [13], [14], [15], [16], [17], [18], [19]. The main characteristics of these studies can be found in Table 1.

**Table 1:** Basic study characteristics.

Author, year	Age (Y), sex	Symptoms	EBV diagnostic serology	Initial amylase (U/L)	Initial lipase (U/L)	Pancreatitis diagnostic imaging	Complications	All treatments administered	Outcome	Other potential contributors	Etiologies ruled out
Accomando, S., et al. [3]	3, F	Abdominal pain, vomiting	VCA IgM	913	6450	US: enlarged pancreas with hypoechogenic areas	None mentioned	Cefotaxime, fasting, parenteral nutrition, analgesics	Recovery	None mentioned	Gallstones, trauma, medications, hereditary, other viruses
Bar, R. S., et al. [8]	18, F	Malaise, nausea, vomiting, shortness of breath	VCA IgM EA-D IgG	8500	N/A	X-ray: upper abdominal mass between the stomach and colon	Acute hepatitis with clotting factor deficiency, acute myocarditis, acute renal failure, shock	Corticosteroids, digitalis, antibiotics, insulin, intranasal oxygen, IV sodium bicarbonate, hemodialysis	Recovery	None mentioned	Other viruses
Coffin, M. K., et al. [9]	25, M	Severe epigastric pain radiating to the back	EBNA VCA IgM	N/A	> 10,000	MRI: diffuse inflammation of the tail of the pancreas	Recurrence of acute pancreatitis	Fluid resuscitation and pain management	Recovery	None mentioned	Alcohol use, triglycerides, other viruses, gallstones, hereditary
Descamps, V., et al. [10]	40, M	Generalized skin eruption	EA-D IgM EA-D IgG EBV DNA PCR	1070	280	CT: increase of the size of the pancreas compatible with an acute oedematous pancreatitis without necrosis	Staphylococcal bacteremia	Amoxicillin, no further specifications	Recovery	Drug-induced hypersensitivity syndrome following a 3-week course of allopurinol Positive HHV-6 IgM	Other viruses
Galzerano, A., et al. [11]	3, F	Abdominal pain, respiratory distress	EBV DNA PCR in pleural fluid	3880	N/A	CT: enlargement of the pancreatic head with Wirsung's duct ectasia, lack of opacification of a segment of the extrahepatic portal vein, up to the junction with the splenic vein and superior mesenteric artery with evidently swollen peripancreatic circles	Bilateral pneumonia, left pleural effusion	Thoracentesis, decortication, meropenem, teicoplanin, total parenteral nutrition, ganciclovir	Recovery	History of celiac disease	None
Hammami, M. B., et al. [12]	18, F	Sore throat, headache and malaise followed by upper abdominal pain, nausea and anorexia.	EBNA VCA IgM	327	2016	CT: acute pancreatitis with mild splenomegaly	Hepatosplenomegaly	Pain management, hydration, fluids and slowly advanced diet.	Recovery	None mentioned	Gallstones, other viruses, autoimmune
Huang, L., et al. [13]	45, F	Dry cough, chest discomfort, abdominal pain	EBV IHC staining on gastric ulcer biopsy EBV DNA PCR	> 3 times ULN	> 3 times ULN	CT: sausage-like and obviously swollen pancreas, uneven density of the pancreatic parenchyma with large volumes of reduced enhancement. Severe, minimally exudative pancreatic body necrosis wrapped within capsule-like rim. Moderate pericardial effusion and mild pleural effusion on the left side.	Gastritis with gastric ulcer, pericarditis	Supportive treatment (no further specification)	Multiple organ failure, death	History of resected thymoma	None
Kang, S. J., et al. [14]	11, F	Left upper quadrant abdominal pain, nausea and vomiting	EBNA VCA IgM	4010	4941	CT: swelling of the pancreas with peripancreatic fluid accumulation compatible with acute pancreatitis	Acute hepatitis	Fasting, total parenteral nutrition	Recovery	None mentioned	Trauma, gallstones, hereditary, medications, other viruses, autoimmune.
Khawcharoenporn, T., et al. [15]	18, F	Sore throat, vomiting, abdominal pain, and decreased appetite	VCA IgM Immuno-phenotyping of bone marrow lymphocytes	620	659	CT: moderate gallbladder wall thickening, pericholecystic fluid, edematous pancreas, and hepatosplenomegaly	Shock, DIC, hepatosplenomegaly, acalculous cholecystitis	Aztreonam, clindamycin, moxifloxacin, supportive treatment (no further specification)	Recovery	None mentioned	Gallstones, other viruses.
López-Ibáñez, M. C., et al. [16]	15, M	Epigastric pain, referring to the back	Only EBV heterophile IgM	1251	N/A	CT: globular pancreas, mild hepatosplenomegaly, moderate ascites and significant left paraaortic lymphadenopathy	Tonsillitis, hepatosplenomegaly	Not specified	Not specified	None mentioned	Gallstones
Marín-García, D., et al. [17]	15, F	Odynophagia, followed by severe epigastric abdominal pain radiating to the back and nausea	VCA IgM	N/A	2930	N/A	None mentioned	Paracetamol, ibuprofen, no further specification	Recovery	Ibuprofen use	Gallstones, triglycerides
Singh, S., et al. [18]	21, F	Malaise, epigastric pain radiating to the back, nausea, vomiting and sore throat	VCA IgM	N/A	4301	CT: pancreatic edema and peripancreatic stranding, consistent with acute pancreatitis, as well as periportal lucency with mild pericholecystic edema and mild splenomegaly	AIHA, splenomegaly	Prednisone, blood transfusions, conservative management (no further specification)	Recovery	Occasional drinking (alcohol), but the last drink was 10 days prior to admission	Other viruses, autoimmune.
Teniente Urbina, M. E., et al. [19]	39, F	Odynophagia and diarrhea followed by severe abdominal pain and repeated vomiting	EBNA VCA IgM	195	212	CT: acute pancreatitis, Balthazar grade B.	Pneumonia, acute myocarditis leading to left-sided HFrEF with pulmonary edema, respiratory failure, interstitial nephritis, distributive shock	Azithromycin, oral corticosteroids, omeprazole, antacids, broad-spectrum antibiotics, inotropes, intubation with mechanical ventilation, hemodialysis	Recovery	Laparoscopic surgery for endometriosis, 2-day course of IV ketorolac	Other viruses
Our case	40, M	Sore throat, abdominal pain	VCA IgM	N/A	3704	CT: non-complicated acute pancreatitis	Peritonsillar abscess	Ceftriaxone, metronidazole, dexamethasone, incision and drainage, clindamycin, fasting, intravenous nutritional support and analgesics	Recovery	Regular alcohol consumption (∼7 drinks / week), moderately elevated triglycerides (4.13 mmol/L), 8-month course of semaglutide use	Gallstones, other viruses

Abbreviations: Y = years; EBV = Epstein-Barr virus; U/L = Units per liter; F = Female; VCA = Viral capsid antigen; IgM = Immunoglobulin M; US = Ultrasound; EA-D = Early antigen D; IgG = Immunoglobulin G; N/A = Not applicable; IV = Intravenous; M = Male; EBNA = Epstein-Barr Nuclear Antigen; MRI = Magnetic resonance imaging; DNA = Deoxyribonucleic acid; PCR = Polymerase chain reaction; CT = Computed tomography; DIC = Disseminated intravascular coagulation; IHC = Immunohistochemistry; ULN = Upper limit of normal; AIHA = Autoimmune hemolytic anemia; HFrEF = Heart failure with reduced ejection fraction.

The oldest included study was published in 1975, while the newest appeared in scientific journals as recently as in the year 2025. 8 cases pertained to the adult population, while 5 cases related to children and adolescents under the age of 18. Most patients were female (10 out of 13). The most frequently mentioned symptom was epigastric / abdominal pain, present in 11 out of 13 cases, with 4 specifying pain radiation towards the back. Only 4 out of 13 studies reported typical mononucleosis-associated throat soreness (odynophagia).

The most frequently utilised serological diagnostic methods for acute EBV infection / reactivation detection were the viral capsid antigen (VCA) IgM assays, done in 9 out of 13 cases. In all cases, either amylase, lipase or both were dosed; the results were variable, ranging from 195 to 8500 U/L for amylase, and 212 to > 10,000 U/L for lipase on initial bloodwork. In most cases, computed tomography (CT) was the imaging modality of choice for detection of acute pancreatitis (9 out of 13 studies), with ultrasound often performed concomitantly to eliminate the presence of a biliary etiology. In one case, CT scanning did not detect acute pancreatitis, but clinical suspicion and recurrence of typical pancreatitis-associated pain prompted the physicians to perform magnetic resonance imagery (MRI), which revealed diffuse inflammation of the pancreatic tail [9].

A wide variety of complications were noted in the included reports, such as tonsillitis, hepatosplenomegaly, hepatitis, myocarditis, gastritis, interstitial nephritis, pneumonia, disseminated intravascular coagulation (DIC), autoimmune hemolytic anemia (AIHA) as well as shock. Aside from treatment of these additional complications, the management of EBV-associated pancreatitis was always conservative, most often involving fasting, IV rehydration, total parenteral nutrition and analgesia. In one case, the antiviral medication ganciclovir was also used [11]. Only one of the included cases resulted in the death of the patient following multiple organ failure [13]. All other patients made complete recoveries without any obvious long-lasting sequelae.

While most cases did not involve any other clear causes for the occurrence of acute pancreatitis, a few studies highlighted additional risk factors that may have contributed to the pathogenesis of the disease. Examples included occasional alcohol consumption, allopurinol use with subsequent drug-induced hypersensitivity syndrome, co-infection by human herpesvirus 6 (HHV-6), history of auto-immune disorders, non-steroidal anti-inflammatory drug (NSAID) use, recent abdominal surgical intervention and history of resected thymoma.

## 3 Discussion

Acute pancreatitis can arise from multiple etiologies. Pathogenesis generally involves premature pancreatic enzyme activation, calcium signaling disruption, mitochondrial dysfunction, endoplasmic reticulum stress, impaired autophagy, acute inflammation, cell death, oxidative stress, and microcirculatory dysfunction [20]. Clinical diagnosis usually requires at least two of the following: (i) abdominal pain consistent with pancreatitis, (ii) serum amylase or lipase levels > 3 times the upper limit of normal, (iii) typical findings on abdominal imaging (CT or MRI) [21].

Based on our review, EBV-associated pancreatitis presents heterogeneously, sometimes without abdominal pain or classic mononucleosis features. While most cases follow a benign course and resolve fully, we identified one fatal case [13]. That patient had a prior thymoma resection, a condition that can cause immunodeficiency and predispose to recurrent, severe or fatal viral infections, as reported in other cases [22], [23]. Although EBV-associated pancreatitis typically resolves spontaneously, it is often accompanied by other organ complications such as hepatitis, gastritis, myocarditis, or interstitial nephritis. Some authors have speculated that antiviral therapy could reduce EBV-related complications; however, current evidence is insufficient, and no medication has been approved for the treatment of EBV infection to date [24].

In this review, specific recovery times were not analyzed due to inconsistent reporting across cases. Many reports lacked precise timelines for symptom resolution or enzyme normalization, making comparisons unreliable. For this reason, outcomes were summarized simply as “recovered” when stated. In fact, among the 13 included cases, only one report by Kang *et al*. explicitly documented the time to enzyme normalization, noting that serum levels returned to normal after 4 days [14]. When looking at the broader literature, it seems that serum lipase in uncomplicated acute pancreatitis typically normalizes within approximately 8–14 days, although variation exists depending on disease severity [25], [26].

Determining definitive EBV causality for pancreatitis is quite challenging. Indeed, the gold-standard test would likely be in situ hybridization for EBV-encoded RNA on pancreatic tissue, requiring a biopsy which is not part of standard care in suspected viral pancreatitis [27]. Consequently, diagnosis mainly relies on fulfillment of diagnostic criteria for acute pancreatitis, evidence of acute EBV infection on serology and absence of other common etiologies for acute pancreatitis.

In our patient, pancreatitis was likely related to EBV, but several other factors could be considered. Alcohol consumption is the second most common cause after gallstones, acting through mechanisms such as intracellular calcium increase, transcription factor activation, stellate cell stimulation, and pro-inflammatory cytokine release [28], [29]. Our patient had about 7 drinks weekly, whereas alcoholic pancreatitis is usually linked to > 5 drinks daily [28]. He also had moderately elevated triglycerides (4.13 mmol/L, ≈366 mg/dL). Hypertriglyceridemia can cause pancreatitis through ischemia, acidosis, and toxic free fatty acids [30], but this typically occurs at levels > 11.3 mmol/L (1000 mg/dL), with 4.6 mmol/L (400 mg/dL) generally considered the lower threshold [31]. Drug-induced pancreatitis is also a possible cause [32]. Our patient used semaglutide and pantoprazole chronically. Although GLP-1 agonists have been hypothesized to cause pancreatitis, with plausible mechanisms being proposed in murine models, pooled human data show no significant association [33], [34]. As for pantoprazole, some studies even suggest improved outcomes in pancreatitis [35].

It is reasonable to consider that some of these factors, while individually sub-pathogenic, may act synergistically to lower the threshold for pancreatitis, allowing EBV to precipitate disease. Prior studies support this “threshold model,” in which protective and stressor factors interact until a critical load is reached [36]. Preventive strategies should therefore focus not only on controlling dominant risk factors but also on managing multiple minor contributors that may act in concert.

Concerning the risk of recurrence of EBV-associated pancreatitis, there does not seem to be any literature specifically addressing the topic; only one of our included cases reported a recurrence in disease [9]. More general evidence on EBV recurrence suggests that reactivation is uncommon in immunocompetent individuals and is primarily associated with immune system compromise [37]. By extension, recurrence of EBV-associated pancreatitis would also be expected to be rare, although this remains speculative given the limited number of reported cases and warrants further investigation.

As for long-term pancreatic function following EBV-associated pancreatitis, most included reports described the disease as a self-limiting condition with full clinical recovery. None of the studies performed specific long-term assessments of pancreatic endocrine or exocrine function, and follow-up data were generally limited to short-term clinical outcomes. General acute pancreatitis literature indicates that both endocrine and exocrine dysfunction can occur after an episode of acute pancreatitis, particularly in cases of severe disease [38]. While most EBV-associated cases appear to be mild, the absence of systematic long-term evaluation makes it difficult to determine whether subtle or delayed pancreatic impairment occurs in this population.

When compared to other viral etiologies of pancreatitis, EBV appears to be a relatively uncommon cause. A large systematic review published in 2021 identified hepatitis viruses, coxsackieviruses, and hemorrhagic fever viruses as the most frequent viral triggers, with cytomegalovirus associated with the highest number of fatal cases. In contrast, Epstein–Barr virus accounted for far fewer reported cases and, historically, none of the deaths attributed to viral pancreatitis, with the first fatal case described only more recently in 2022 [39]. These findings suggest that Epstein–Barr virus is both a less common and generally less severe viral cause of pancreatitis when compared with other pathogens. This review is mainly limited by the small number of published cases. Publication bias is also likely, as atypical or severe cases are more frequently reported than mild or self-limited episodes. These limitations restrict the ability to draw more firm conclusions about causality, risk factors, and management.

Further studies are needed before high-quality quantitative research on EBV-associated pancreatitis is possible. Future reports should provide transparency about potential coexisting risk factors, clarifying whether EBV acts more often as an opportunistic trigger in susceptible patients or as an independent cause. Even with such efforts, unrecognized genetic predispositions may remain an unavoidable limitation in understanding this rare disease.

## Author agreement statement

We confirm that all authors made significant contributions to this work, have reviewed and approved the final manuscript, and agree to its submission. The manuscript is original, has not been published previously, and is not under consideration elsewhere.

## CRediT authorship contribution statement

**Gilbert Cornut:** Writing – review & editing. **Joe Zako:** Writing – original draft, Visualization, Validation, Project administration, Methodology, Investigation, Formal analysis, Data curation, Conceptualization. **Alexandra Monière-Wollank:** Writing – review & editing, Validation, Supervision, Methodology, Conceptualization.

## Funding

This research did not receive any specific grant from funding agencies in the public, commercial, or not-for-profit sectors.

## Declaration of Competing Interest

The authors declare no conflicts of interest.
